# Investigation of MAO Coatings Characteristics on Titanium Products Obtained by EBM Method Using Additive Manufacturing

**DOI:** 10.3390/ma15134535

**Published:** 2022-06-28

**Authors:** Sergey Grigoriev, Nikita Peretyagin, Andrey Apelfeld, Anton Smirnov, Oleg Yanushevich, Natella Krikheli, Olga Kramar, Sergey Kramar, Pavel Peretyagin

**Affiliations:** 1 Spark Plasma Sintering Research Laboratory, Moscow State University of Technology “STANKIN”, Vadkovsky per.1, 127055 Moscow, Russia; s.grigoriev@stankin.ru (S.G.); p.peretyagin@stankin.ru (P.P.); 2 Scientific Department, A.I. Evdokimov Moscow State University of Medicine and Dentistry, Delegatskaya Street 20, Build 1, 127473 Moscow, Russia; olegyanushevich@mail.ru (O.Y.); nataly0088@mail.ru (N.K.); dr.ovkramar@gmail.com (O.K.); kramarsv@mail.ru (S.K.); 3 Department 1203, Moscow Aviation Institute (National Research University), Volokolamskoe Shosse, 4, 125993 Moscow, Russia; apelfeld@yandex.ru

**Keywords:** titanium alloy Ti-6Al-4V, additive manufacturing, electron beam melting, micro-arc oxidation, MAO coating, thickness, roughness, surface morphology, structure, composition, heat resistance, thermal cycling test

## Abstract

Coatings with a thickness from 27 to 62 µm on electron beam melted Ti-6Al-4V have been formed by micro-arc oxidation (MAO) in a silicate-hypophosphite electrolyte. MAO tests in the anode-cathode mode (50 Hz) with an anode-to-cathode current ratio of 1:1 and sum current densities 10 and 20 A/dm^2^ were carried out. The duration of the MAO treatment was 30 and 60 min. The effect of the processing parameters on the structural properties of the MAO treated coatings was studied. The current density and treatment time significantly affect the coating thickness and surface roughness. The values of these characteristics increase as the current density increases. The effect of thermal cycling tests on surface morphology, thickness and roughness, and elemental and phase composition of MAO coatings was analyzed. After 50 cycles of thermal cycling from +200 °C to −50 °C, no cracking or delamination of coatings was observed. Coatings formed in 30 min at a current density of 20 A/dm^2^ turned out to be the best in terms of such indicators as surface morphology, thickness, and roughness.

## 1. Introduction

Metal alloys are the most common materials used for manufacturing implants because their mechanical properties meet the load bearing requirements. Three alloys are widely used in the production of joint prostheses: stainless steel, cobalt-chromium, and titanium alloys. Commercially pure titanium and titanium alloy Ti-6Al-4V are the most commonly used for bone replacements. The use of Ti-6Al-4V alloy as a surgical material began in the early 1960s after it was found to have a high osseointegration capability. This (α + β)-alloy with a relatively low density has good strength and ductility with high crack resistance [1,2].

Traditional methods for producing metal products, such as casting, pressure treatment and cutting, are associated with certain difficulties in the case of titanium alloys. Currently, mass production of medical implants is carried out using multi-axis machining on machine tools with numerical control. The low productivity of such processing and the high cost of instruments increase the final price of implants. In addition, patients with abnormal anatomy require implant designs that correspond to the individual characteristics of the body [3,4].

Additive manufacturing is an innovative process, the essence of which is to obtain products of almost any shape without subsequent mechanical processing, which in particular leads to a reduction in waste. This is achieved by layer-by-layer formation of the product from the powder. Its particles are sintered and fused by local heating by a laser or electron beam scanning the surface of a thin layer of powder deposited on a pre-prepared substrate. With this technology, in contrast to machine processing, the product is created for one pass, and the input data is its computer model [5,6].

For additive manufacturing of products from titanium alloys, the most commonly used methods are selective laser melting (SLM) and electron beam melting (EBM) using appropriate powders. For the additive manufacturing of reactive metals and alloys such as titanium alloys, the EBM method is more suitable due to the possibility of using “vacuum printing”. In addition, the EBM method provides a higher throughput (80 cm^3^/h) compared to the SLM method (20–40 cm^3^/h), and also uses a wider range of powder particle sizes—45–105 µm versus 20–45 µm for the SLM method [7,8]—that increases the availability of the EBM method. Another important advantage is that products made of titanium alloys obtained by the EBM method usually do not require subsequent heat treatment to relieve residual stresses due to high temperatures in the powder layer sintering zone.

However, unlike bioactive ceramics, bioglass, hydroxyapatite, and glass ceramics, titanium implants cannot effectively contact bone due to their low osseointegration and osteoinductive properties. This requires a certain modification of the implant surface, including the application of appropriate coatings. The chemical composition, hydrophilicity, morphology, and surface roughness in this case are key factors for the active interaction of the implant with bone tissue and subsequent osseointegration [9,10]. Surface modification methods such as plasma spraying [11,12,13,14,15], alkali treatment [16,17,18,19,20], acid etching [21,22,23,24], laser treatment [25,26,27,28], and anodizing [29,30,31] are used, as well as the increasingly widely used plasma-electrolytic method of microarc oxidation (MAO) [32,33,34,35,36]. MAO is commonly used to produce useful coatings with various oxide layers on the surface of various metals, such as Ti, Al, Mg and Zr and their alloys, to improve corrosion resistance, tribological properties, biocompatibility and thermal properties [37,38,39,40,41,42,43].

Compared with others, MAO is one of the most effective methods of surface modification due to the formation of a porous bioceramic layer on the surface of titanium and its alloys. In the MAO process, the specimen is immersed as one of the electrodes in an electrolyte bath (which can serve as a counter electrode) with an aqueous solution of the corresponding salts (silicates, phosphates, etc.) or a suspension containing suspended dispersed particles of the necessary compounds (oxides, silicides, etc.) [44]. The formation of MAO coatings occurs both due to the oxidation of the material of the treated substrate, and due to the incorporation of compounds or particles contained in the electrolyte into the coating. The characteristics of the resulting coatings are affected by both the nature of the base material and the technological parameters of the MAO process, such as the composition and temperature of the electrolyte, the electrical parameters of the mode, and the duration of treatment, which can vary over a wide range [32].

Considerable work has been devoted to the study of changes in surface characteristics, corrosion, and tribocorrosion behavior of pure Ti and alloy Ti-6Al-4V treated by the MAO process. For example, Khanmohammadi et al. reported that higher coating thickness, lower percentage of surface porosity, and smaller pore diameter led to higher corrosion resistance of coatings on titanium alloy Ti-6Al-4V [37]. Shokouhfar et al. demonstrated that the addition of SiC, Al_2_O_3_, and TiO_2_ nanoparticles to the aluminate electrolyte improved the corrosion and wear resistance of MAO coatings formed on the titanium substrate [38]. Teng et al. showed that the applied voltage plays an important role in the characteristics and properties of the composite coatings on the titanium substrate. With the increase of the applied voltage, the pore size, surface pore density, and the roughness of the coatings increase, while the corrosion resistance decreases [39]. Fazel et al. investigated the effect of MAO coatings on pure titanium and alloy Ti-6Al-4V at 180 V, and the results indicated that, unlike the volcanic morphology of oxide layer on pure Ti, a cortex-like morphology with irregular vermiform slots was seen on the MAO/Ti-6Al-4V samples, and a lower wear volume loss was achieved for these samples [40]. Therefore, one of the advantages of this method is that porous oxide coatings on titanium alloys obtained by the MAO technique have enhanced wear and corrosion resistance with the desired bioactivity [41,42,43].

However, there are no data in the scientific literature that allow us to recommend microarc oxidation for the treatment of biomedical products made of titanium alloys by the EBM method using additive technology.

In temperature applications, the thermal stability of the MAO coatings is a critical parameter. However, very little information is available with respect to their thermal fluctuations in research papers. On the other hand, considering the possibility of using these materials in medicine, it should be noted that all medical devices should be sterile when used because any microbial contamination could result in infection transmission. The steam sterilization, also known as autoclaving, is one of the most utilized technologies because it is reliable, consistent, and lethal to microorganisms, while also being safe for the staff who operate the autoclave. The upper limit of the temperature range (+200 °C) was chosen just above the temperature specified in the standard for steam sterilization and sterility assurance in health care facilities [45]. Therefore, all experiments have been carried out in a steam atmosphere in order to simulate the autoclaving method. The lower limit (−50 °C) was chosen from climatic considerations.

The purpose of this work was to study the effect of thermal cycling on the surface morphology, thickness and roughness, and elemental and phase composition of MAO coatings obtained on samples made of Ti-6Al-4V titanium alloy powder by electron beam melting.

## 2. Materials and Methods

The chemical composition of Ti-6Al-4V powder with an average particle size of d_50_ = 70 ± 0.05 μm supplied by Arcam AB (Mölndal, Sweden), which was used for fabricating the substrates, is presented in Table 1. Rectangular samples with a size 28 mm × 19 mm × 5 mm were fabricated by the EBM method using additive technology on the Arcam A2 machine (ARCAM AB, Mölndal, Sweden) according to the following scheme. On a stainless-steel platform preheated to 750 °C, layers of powder about 50 µm thick were successively applied. The electron beam speed was varied from 240 mm/s to 1200 mm/s. The average power of the electron beam and vacuum were 900 W and 0.2 kPa, respectively. All of the produced samples were polished to 4000 grit paper and washed in acetone for about 15 min in an ultrasonic bath. The density of obtained samples was determined by the choice of electron beam parameters and scanning speed values, and was ~99.6% relative to the theoretical one.

MAO treatment was carried out in a silicate-hypophosphite electrolyte with pH~9 comprised of 12.5 g·L^−1^ of Na_2_SiO_3_∙9H_2_O and 5 g·L^−1^ of Na(PH_2_O_2_)∙H_2_O on an experimental setup in the anode-cathode mode (50 Hz), with an anode-to-cathode current ratio of 1:1 and sum current densities 10 and 20 A/dm^2^. It should also be noted that sodium hypophosphite Na(PH_2_O_2_) is a reducing agent, and at the initial stage of the MAO process in the anode-cathode mode, it can facilitate the ignition of the discharge. Further, when heated in the zone of the discharge channels, it decomposes (at a temperature above 230 °C) with the formation of sodium pyrophosphate Na_4_P_2_O_7_, which, like sodium silicate Na_2_SiO_3_, which is also a part of the electrolyte, is a corrosion inhibitor, stimulating the formation of oxide layer. The duration of the MAO treatment was 30 and 60 min. The samples code and the main technological parameters of the MAO process are presented in Table 2.

The morphological analysis and elemental composition of the substrates have been studied with a G2 Pro desktop scanning electron microscope combined with a detector for chemical characterization (Phenom, Waltham, MA, USA). The surface morphology and cross-sectional structure of MAO coatings were studied using the scanning electron microscope (SEM) VEGA 3 LMH (Tescan, Brno, Czech Republic) equipped with an energy dispersive X-ray spectrometer (EDS). The thickness of MAO coatings was measured with an eddy current thickness gauge (KID, Moscow, Russia). For each sample, 15 measurements were carried out. The roughness was measured using the optical system MicroCAD premium+ (GFM, Berlin, Germany). X-ray phase analysis was carried out in Co Kα radiation at a voltage of 30 kV and angles 2θ from 25 to 80 degrees on the X-ray diffractometer Difrey-401k (Scientific Instruments, St. Petersburg, Russia).

In order to evaluate the thermal stability of MAO coatings and the effect of thermal cycling on their surface morphology, thickness and roughness, and elemental and phase composition of the coatings, thermal cycling tests (TCT) were carried out on a Thermotron ATSS-30-2-2 (Thermotron, Holland, MI, USA). Obtained samples were first loaded into the hot chamber, heated to +200 °C, kept in it for 10 min, and then transferred to the cold chamber with a temperature of −50 °C, where the samples were kept for another 10 min. The test program included 50 heating and cooling cycles.

## 3. Results and Discussion

**Figure 1:**
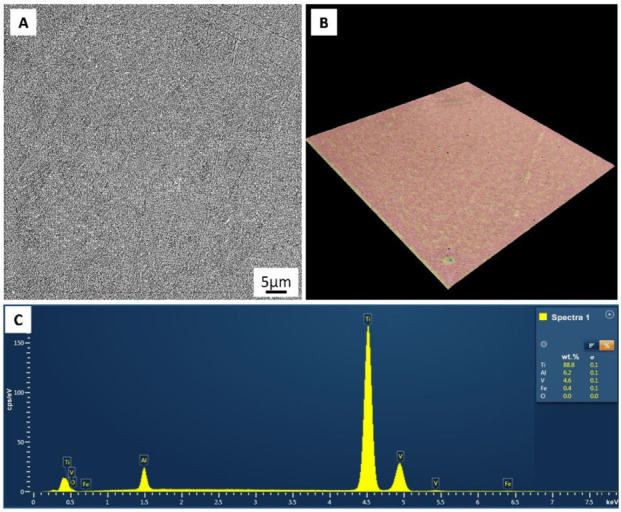
Substrate surface morphology (**A**), profile of the substrate surface (**B**), and elemental analysis of the surface of substrate (**C**).

A representative electron microscope image of the microstructure, the surface morphology, and the EDS analysis of the substrate are depicted in Figure 1. The structure of the Ti-6Al-4V melted by the electron beam consists of columnar β grains which are differentiated by α grains and lamels united in colonies (Figure 1A). The profile of the substrate surface has a homogeneous poreless structure (Figure 1B). The elemental composition of the fabricated samples corresponds to the chemical composition of Ti-6Al-4V powder (Figure 1C).

**Figure 2:**
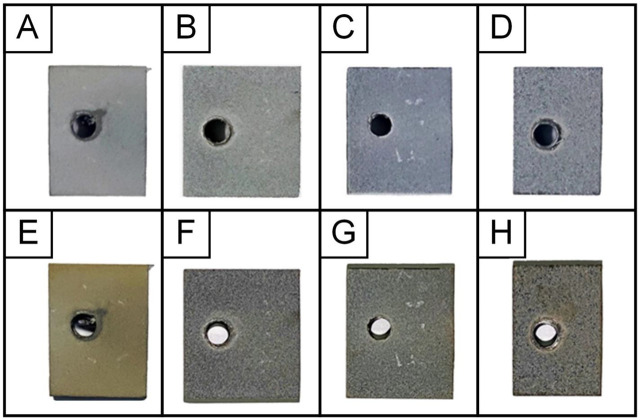
Samples with MAO coatings Ti-MAO-10-30 (**A**,**E**), Ti-MAO-10-60 (**B**,**F**), Ti-MAO-20-30 (**C**,**G**), and Ti-MAO-20-60 (**D**,**H**), before (**top row**) and after (**bottom row**) TCT.

As a result of thermal cycling tests, it was found that all samples with MAO coatings withstood 50 heating and cooling cycles without cracking and exfoliation of coatings. Figure 2 shows the appearance of samples before and after TCT with coatings obtained at various technological parameters of the MAO process. It can be seen that the Ti-MAO-10-60 and Ti-MAO-20-30 samples look best: they turned the least yellow after TCT due to thermal oxidation of the titanium base (titanium dioxide—anatase is characterized by a greenish-yellow tint), due to access of oxygen to it via through pores in the coating when heated in air. Service holes on coated samples are used to secure current leads to them for immersion into the electrolyte (Figure 2).

**Figure 3:**
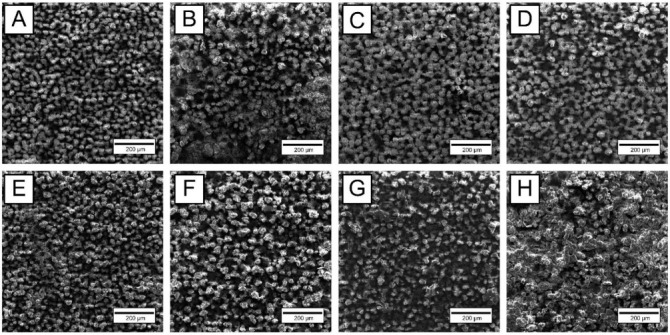
SEM images of surface morphology: MAO coatings Ti-MAO-10-30 (**A**,**E**), Ti-MAO-10-60 (**B**,**F**), Ti-MAO-20-30 (**C**,**G**), and Ti-MAO-20-60 (**D**,**H**), before (**top row**) and after (**bottom row**) TCT.

Figure 3 shows SEM images of the surface morphology of MAO coatings obtained at various technological parameters of the MAO process, before and after TCT. It can be seen that on the Ti-MAO-10-30 and Ti-MAO-10-60 samples, the oxide “stalagmites” that have grown during MAO on a titanium base are not yet sufficiently consolidated, and on the Ti-MAO-20-60 sample, the coating after TCT becomes too rough and heterogeneous. The best morphology of coating surface is observed on the Ti-MAO-20-30 sample, which is probably due to successful combination of temperature-time characteristics in microarc discharges at sum current densities 20 A/dm^2^ and MAO treatment duration 30 min. At the same time, the open porosity of the MAO coating on the Ti-MAO-20-30 sample looks sufficient both in terms of pore size and “percentage” of porosity for the possibility of bone tissue ingrowth into the coating.

Table 3 presents the results of measuring the thickness and roughness of MAO coatings obtained at various technological parameters of the MAO process, before and after TCT. It can be seen that oxidation reduces the initial roughness of the metal surface, especially at the initial stage of the MAO process. After TCT, the thickness of coating on the Ti-MAO-10-30 sample almost halved, which may indicate that thin oxide “stalagmites”, that were almost not consolidated (not sintered) with each other, during thermal cycling cracked almost at half their height and fell off. In this case, the coating thickness decreased from 27 to 15 μm. With increasing duration of MAO treatment and current density, the thickness of the coatings increased, and the bases of the oxide “stalagmites” coalesced into an almost continuous layer. During thermal cycling, only their tops broke off, and the thickness of the MAO coatings decreased insignificantly.

Table 4 presents the elemental composition of the surface layer of MAO coatings obtained at various technological parameters of the MAO process, before and after TCT. It can be seen that, regardless of the technological parameters of the MAO process, the surface layer of coatings contains several times more silicon than titanium. This indicates that this layer is formed mainly due to the components of the electrolyte, and not the base metal.

**Figure 4:**
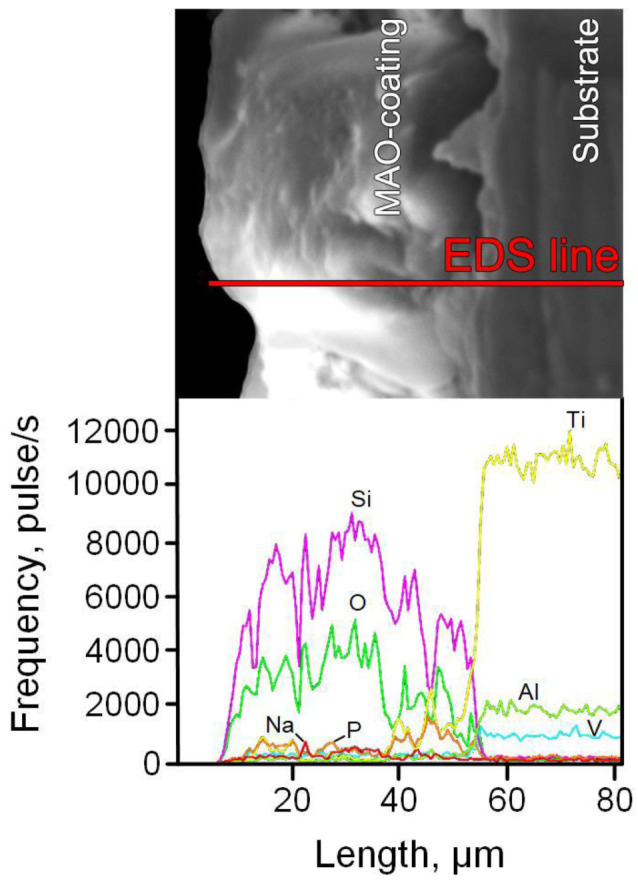
The cross-sectional microstructure of MAO coating (Ti-MAO-20-30_bT), and the element distribution along the marked line.

In the inner layer of the MAO coating, there is much less silicon and much more titanium (Figure 4).

As for the alloying elements of the Ti-6Al-4V alloy, with an increase in the current density and the duration of MAO treatment, the aluminum content in the surface layer of the coatings increases significantly, while the vanadium content decreases (Table 4). It can be seen that there is not enough oxygen for all titanium and silicon in the coating to be in the form of stoichiometric dioxides TiO_2_ and SiO_2_. This indicates that titanosilicates and aluminosilicates may be present in the coating (Figure 4). After TCT, the content of all elements in the coating, except for aluminum, remains approximately at the same level as before TCT (Table 4). For aluminum, except for sample Ti-MAO-10-30, an almost two-fold increase in its content after TCT is observed, which may be due to the lower current density and treatment time of MAO compared to other samples (Table 4).

**Figure 5:**
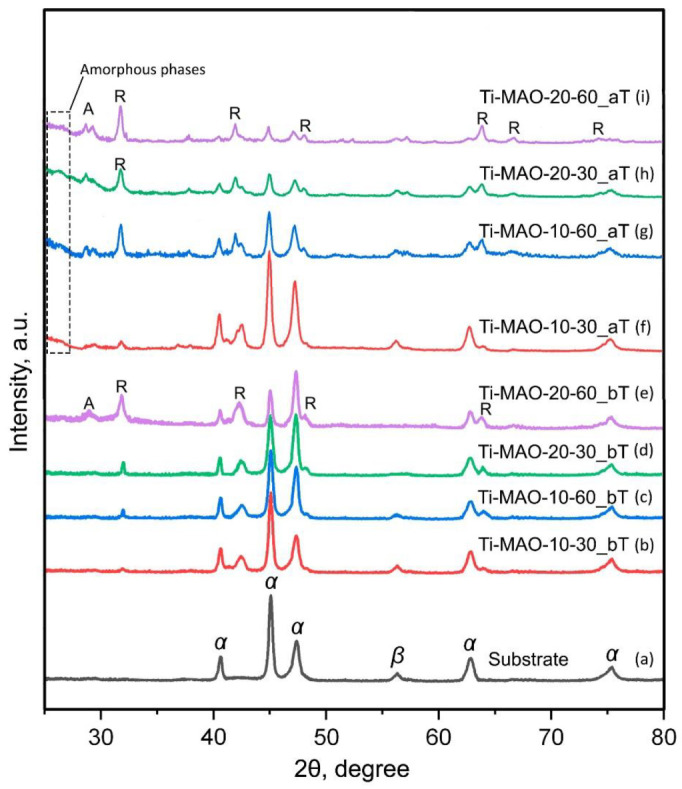
X-ray diffraction patterns of substrate (**a**) and samples with MAO coatings Ti-MAO-10-30 (**b**,**f**), Ti-MAO-10-60 (**c**,**g**), Ti-MAO-20-30 (**d**,**h**), and Ti-MAO-20-60 (**e**,**i**), before (**b**–**e**) and after (**f**–**i**) TCT. A—α-Ti; β—β-Ti; A—TiO_2_-anatase; R—TiO_2_-rutile.

Figure 5 shows the X-ray diffraction patterns of the metal base and MAO coatings obtained at various technological parameters of the MAO process, before and after TCT.

It can be seen that, in the base, manufactured by the EBM method using the additive technology from the powder of the titanium (α + β) alloy Ti-6Al-4V, there are α-Ti and β-Ti phases. After MAO treatment in Ti-MAO-10-30, Ti-MAO-10-60, and Ti-MAO-20-30 coatings, X-ray phase analysis identifies titanium dioxide only in the TiO_2_-rutile modification. This is explained by the fact that another modification of titanium dioxide, TiO_2_-anatase, when heated to 620–650 °C, turns into rutile, which is denser and harder. Such heating is provided by the plasma of microdischarges migrating over the treated surface. In addition to rutile, anatase is present in the Ti-MAO-20-60 coating with a thickness of more than 60 μm, which may indicate the “immersion” of the high-temperature discharge region deep into the pore channels of MAO coating, and insufficient heating of its surface layer. After thermal cycling tests, the X-ray diffraction patterns of all coatings show the TiO_2_-anatase phase, which can be explained by the thermal oxidation of titanium when the substrate is heated and air penetrates to it through pores in the MAO coating. It is known [46] that during aging, titanium dioxide obtained from an alkaline medium (the pH of the silicate-hypophosphite electrolyte is significantly more than 7) partially becomes X-ray amorphous. In addition, the absence of peaks of SiO_2_ (α-cristobalite and α-quartz) at large amount of silicon in the surface layer of MAO coatings (Table 4), as well as titanosilicate and aluminosilicate peaks on the diffractograms, indicate that they are in an amorphous state. The amorphization of the oxide layer is indicated by the presence of a “halo” in diffraction patterns (Figure 5f–i) at small angles of 2θ (less than 30 degrees).

## 4. Conclusions

MAO coatings with a thickness from ~27 to ~62 μm were obtained in silicate-hypophosphite electrolyte of samples made of Ti-6Al-4V titanium alloy powder by the electron beam melting method using additive technology. MAO treatment was carried out in the anode-cathode mode (50 Hz), with an anode-to-cathode current ratio of 1:1 and sum current densities 10 and 20 A/dm^2^. The duration of the MAO treatment was 30 and 60 min. X-ray phase analysis identified titanium dioxide in the MAO coatings, mainly in the TiO_2_-rutile modification. After testing for thermal cycling, the X-ray diffraction patterns of all coatings show the TiO_2_-anatase phase, which can be explained by the thermal oxidation of titanium when the substrate is heated and air penetrates to it through pores in the coating. In addition, after TCT, a “halo” is observed at small angles, which indicates some amorphization of titanium dioxide, which may be associated with its aging during thermal cycling, after which it partially becomes X-ray amorphous. The absence of X-ray peaks of SiO2 at the presence of significant amount of silicon in the surface layer of MAO coatings indicates that silicon dioxide is amorphous. MAO coatings have shown their high thermal stability. When tested for thermal cycling, all samples with coatings withstood 50 cycles of heating to +200 °C and cooling to −50 °C without cracking and exfoliation of the coatings. The combination of the characteristics of surface morphology, thickness, and roughness of the MAO coating, formed in 30 min at a current density of 20 A/dm^2^, proved to be the best.

## Figures and Tables

**Table 1:** Chemical compositions of Ti-6Al-4V powder.

Chemical Compositions, wt%				
Al	V	Fe	O	Ti
6.37	4.12	0.29	0.13	Bal.

**Table 2:** The samples code and main technological parameters of the MAO process.

Samples Code	Electrolyte	Electrical Mode	Sum Current Density, A/dm^2^	Treatment Duration, min
Ti-MAO-10-30	Na_2_SiO_3_∙9H_2_O + Na(PH_2_O_2_)∙H_2_O		10	30
Ti-MAO-10-60			10	60
Ti-MAO-20-30		anode- cathode (50 Hz)	20	30
Ti-MAO-20-60			20	60

**Table 3:** Thickness and roughness of MAO coatings before (bT) and after (aT) TCT.

Sample Code	Thickness, μm	Roughness, μm	
		Ra	Rz
Ti-without MAO		5.6	32.1
Ti-MAO-10-30_bT	27.2	2.3	14.9
Ti-MAO-10-60_bT	42.6	3.7	22.0
Ti-MAO-20-30_bT	42.8	4.3	25.3
Ti-MAO-20-60_bT	66.4	4.9	27.4
Ti-MAO-10-30_aT	15.2	2.2	14.5
Ti-MAO-10-60_aT	36.4	2.7	15.5
Ti-MAO-20-30_aT	38.8	3.3	18.7
Ti-MAO-20-60_aT	61.6	4.3	22.1

**Table 4:** Elemental composition of the MAO coatings surface layer before (bT) and after (aT) TCT.

Sample Code	Element Content, at%						
	O	Na	Al	Si	P	Ti	V
Ti-MAO-10-30_bT	57.82	1.11	0.24	28.98	4.59	6.87	0.39
Ti-MAO-10-60_bT	57.44	1.15	0.31	31.02	3.06	6.72	0.30
Ti-MAO-20-30_bT	57.49	0.84	0.44	31.21	3.12	6.69	0.21
Ti-MAO-20-60_bT	57.68	0.9	0.44	30.56	3.78	6.41	0.23
Ti-MAO-10-30_aT	56.95	1.72	0.27	29.93	4.13	6.58	0.42
Ti-MAO-10-60_aT	56.33	1.63	0.79	30.49	3.15	7.25	0.36
Ti-MAO-20-30_aT	56.27	1.61	0.81	30.52	3.18	7.33	0.28
Ti-MAO-20-60_aT	56.45	2.78	0.87	29.85	2.64	7.16	0.31

## Data Availability

Not applicable.
